# Analysis of Adaptation Mutants in the Hemagglutinin of the Influenza A(H1N1)pdm09 Virus

**DOI:** 10.1371/journal.pone.0070005

**Published:** 2013-07-23

**Authors:** Alicia Jiménez-Alberto, Esmeralda Alvarado-Facundo, Rosa María Ribas-Aparicio, Juan A. Castelán-Vega

**Affiliations:** 1 Departamento de Microbiología, Escuela Nacional de Ciencias Biológicas del Instituto Politécnico Nacional, Distrito Federal, Mexico City, Mexico; 2 Center for Biologics Evaluation and Research, US Food and Drug Administration, Bethesda, Maryland, United States of America; Oak Ridge National Laboratory, United States of America

## Abstract

Hemagglutinin is the major surface glycoprotein of influenza viruses. It participates in the initial steps of viral infection through receptor binding and membrane fusion events. The influenza pandemic of 2009 provided a unique scenario to study virus evolution. We performed molecular dynamics simulations with four hemagglutinin variants that appeared throughout the 2009 influenza A (H1N1) pandemic. We found that variant 1 (S143G, S185T) likely arose to avoid immune recognition. Variant 2 (A134T), and variant 3 (D222E, P297S) had an increased binding affinity for the receptor. Finally, variant 4 (E374K) altered hemagglutinin stability in the vicinity of the fusion peptide. Variants 1 and 4 have become increasingly predominant, while variants 2 and 3 declined as the pandemic progressed. Our results show some of the different strategies that the influenza virus uses to adapt to the human host and provide an example of how selective pressure drives antigenic drift in viral proteins.

## Introduction

Influenza hemagglutinin (HA) is the major glycoprotein component on the surface of the influenza A virus and is responsible for binding sialic acid receptors of target cells and for mediating membrane fusion with the target cell [1]. HA, a homotrimer, is synthesized as an inactive precursor (HA0) that is cleaved by cellular proteases to yield active HA, consisting of the HA1 subunit, responsible for receptor binding, and the HA2 subunit, which is responsible for mediating fusion and is linked by a disulfide bond to the HA1 subunit [2]. Fusion between viral and host cell membranes occurs within endosomes, where low pH induces conformational changes in HA that result in the insertion of the fusion peptide, located at the N-terminus of HA2, into the endosomal membrane. Further conformational changes in HA2 then pull the two membranes together creating a pore through which the viral genetic material can be transferred into the host cytosol [3].

HA can undergo rapid evolution due, in part, to its plasticity, and to the error-prone nature of the virus’s RNA-dependent RNA polymerase, leading to a gradual change in genetic sequences by point mutations. This process is known as antigenic drift, and can result in various phenotypic changes including changes in antigenicity, receptor preference and thereby cell tropism, altered fusion functionality, and virulence. Because the viral genome exists in eight segments, another process known as antigenic shift can occur, whereby new viruses are created when segments from the genome of one virus are incorporated into another virus’s genome, as may occur in a host cell infected by two or more strains. This rapid and large change can give rise to new strains very different from any virus encountered previously, with the potential to cause a pandemic [3].

Because of the ability of influenza viruses to rapidly evolve in response to such selective pressures as herd immunity, the use of drugs for treatment, pressures of adapting to a new host, and complicated epidemiological factors, it is necessary to monitor for changes in the genetic makeup of circulating strains so that annual vaccine strains can be selected, treatment advice can be given, and other public health measures can be planned and implemented (World Health Organization, Global Influenza Programme, http://www.who.int/influenza/en/).

Since its emergence in the spring of 2009, influenza virus A(H1N1)pdm09 has demonstrated many diverse adaptive mutations in the HA protein that have been reported in the gene databases. Of these, only a small number have been extensively studied for their effects on structure and function. An example of one such mutation that has been studied fairly thoroughly is the D222G mutation which has been shown to alter receptor specificity and tropism [4], [5]. D222G has also been shown to be associated with increased virulence [6], though other groups have not seen this association [7], [8] **.** In contrast, for many mutations that have arisen, including S143G, S185T, A134T, D222E, P297S and E374K, there exists little data reported other than epidemiology, phylogeny, and some limited antigenicity studies. Little is known about the effects these mutations have on the structure and function of the HA molecule. For this reason, and because these mutations are located in or near the receptor binding site, major antigenic sites, or near the interface of HA1 and HA2, we have performed molecular dynamic simulations on HA containing one or more of these mutations in order to learn more about how they might alter HA structure and function.

Molecular dynamics simulations are a part of computational chemistry that deal with molecular motions as a function of time. Movement of atoms is dictated by the result of numerical equations that follow Newtońs equations of motion that involve bonded and non-bonded atoms, as well as electrostatic interactions [9]. To better reproduce the movement of molecules, it is necessary to include force fields, which contain energy terms for bonded interactions (bond length, dihedral angles) that have been parameterized to reproduce experimental and quantum-mechanical derived data. Molecular dynamics simulations have allowed the understanding of many biological processes, which are dynamic rather than static mechanisms. Regarding influenza, molecular dynamics simulations have been applied extensively to study the molecular basis of HA binding preference [10]–[12], as well as mutations that affect binding preference [13], [14].

In this study we performed molecular dynamics simulations and ligand-binding free energy analysis on four variants of the HA from the influenza A (H1N1)pdm09 virus; we found that the variants that proliferated as the pandemics progressed had changes in antigenic sites and no significant alterations in ligand binding, while variants that declined had an increased ligand affinity. These findings reveal some of the pathways that this pandemic strain used to disseminate in the human population as a response to selective pressure such as receptor recognition, increased infectivity and evasion of the immune response.

## Methods

### HA Sequences and Multiple-sequence Alignments

Amino acid sequences corresponding to complete HA from influenza virus A(H1N1)pdm09 were downloaded from the OpenfluDB database (http://openflu.vital-it.ch; accessed November 21^st^, 2012) [15]. Sequences were grouped by year of collection and aligned with MAFFT v 7 [16]; consensus sequence and mutation frequencies were calculated with BioEdit v 1.5 [17].

### System Setup for Molecular Dynamics Simulations

We used the glycosylated hemagglutinin structure from the A (H1N1)pdm09 strain (PDB ID 3LZG); to elucidate the effect of mutations in receptor binding, we used PyMOL v 1.2r1 (http://www.pymol.org) to align the structure 2WRG, which contains the human receptor (SIA-(2–6)GAL-(1–4)NAG-(1–3)GAL), with 3LZG, and then merged the coordinates of the human receptor and 3LZG to obtain the HA-receptor complex. Mutations were introduced in the receptor-protein complex with PyMOL, using the most preferred conformer for each residue. The ff03.r1 force field was used for the protein [18], and the GLYCAM06 force field [19] for the carbohydrates. We used the *xleap* module of AmberTools [20] to set up the simulation system. Disulfide bonds were built and the system was solvated in a cube of water (TIP3P model); the limits of the cube were set at 1.2 nm beyond the glycoprotein. The system charge was neutralized with the addition of sodium ions.

### Molecular Dynamics Simulations

We used the Gromacs v 4.0.7 software package [21] for our simulations because it is one the fastest molecular dynamics simulation programs; additionally it contains several useful trajectory analysis programs. Long-range electrostatic interactions were computed by the Particle mesh Ewald (PME) algorithm [22] at each step. All bonds were constrained by the LINear Constraint Solver (LINCS) [23], and Berendsen coupling algorithms [24] were used to maintain constant pressure (1 atm) and temperature (300 K). A 2 fsecond integration step was used to compute all forces. Distance cut offs were set at 1.2 nm for van der Waals forces and 0.9 nm for Coulombic interactions. System coordinates were saved every two picoseconds.

The system was energy-minimized with the steepest descent algorithm (tolerance: 100 kJ mol^−1^ nm^−1^). Then, the solvent was equilibrated around the protein-receptor complex by a short molecular dynamics simulation run of 100 picoseconds at constant temperature and pressure (300K and 1 atm, respectively), restraining the positions of heavy atoms of the complex. After that, restraints were eliminated and the system was simulated for at least 5 nanoseconds at constant temperature and pressure (300K and 1 atm, respectively). Stability of the system was evaluated by root mean square deviation (RMSD) and radius of gyration of protein backbone. Trajectories were visualized with VMD [25], and manipulated and analyzed with several programs included in the Gromacs package (trjconv, g_rms, g_gyrate, g_rmsf, g_hbond, g_distance, g_sasa). Atomic contacts were considered stable if they formed in at least 50% of the frames. Preparation of figures and electrostatic potential calculations were done in PyMOL with APBS [26].

Figures prepared for the manuscript are representative of the system at approximately the 8^th^ nanosecond of simulation for variants 1 and 4; for variants 2 and 3, figures were prepared with the structure at the 4^th^ nanosecond of simulation. Times were chosen to assure that all the studied variants had reached stability; as a further control we verified by visual inspection that those particular snapshots selected for the manuscript were truly representative of the behavior seen in the simulations.

Protein stability was determined with CUPSAT (http://cupsat.tu-bs.de/) [27] and I-Mutant3.0 (http://gpcr2.biocomp.unibo.it/cgi/predictors/I-Mutant3.0/I-Mutant3.0.cgi), which are on-line programs that predict protein stability based on structural data. CUPSAT predicts changes in structural stability based on atom and torsion angle potentials; I-Mutant is a neural-network-based server that predicts protein stability upon point mutations, and was trained on data derived from ProTherm [28], a thermodynamic database for proteins and mutants.

### Binding Free Energy

Binding free energy of protein-receptor complexes was calculated at 10-picosecond intervals with the solvated interaction energy (SIE) method [29]. The SIE function is self-consistent and was optimized with force field terms supplemented by solvation terms:



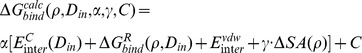
.

Where 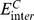 and 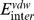 are the intermolecular Coulomb and van der Waals interaction energies in the bound state. 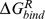 is calculated by solving the Poisson equation with the boundary element method program, BRI BEM, and by using a molecular surface generated with a variable-radius solvent probe. 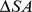 is the change in the molecular surface area upon binding. To solve the function we used sietraj [29], [30], with the default parameters which were derived from a calibration set of 99 protein-ligand complexes: van der Waals radii linear scaling coefficient (ρ = 1.1), the solute interior dielectric constant (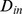 = 2.25), the molecular surface area coefficient (γ = 0.0129 kcal/(mol·Å^2^), a fitting coefficient (α = 0.1048) and a constant (C = −2.89 kcal/mol).

## Results

### Selection of Mutants

We collected complete amino acid sequences from the HA of influenza A(H1N1)pdm09 viruses deposited in the OpenFlu Database. Sequences were grouped by year of collection, and the amino acid frequency for each position was calculated. Due to the large number of sequences collected in 2009 in comparison with the following years, we divided 2009 by semester (January-June had 1158 sequences, whereas July-December had 1607); we grouped the whole number of sequences for 2010 (200), and the last group contained sequences from 2011 until November 2012 (530). We focused on four variants that emerged since the beginning of the 2009 pandemic (Table 1 and Table S1): Variant 1 has two mutations, S143G and S185T. It emerged in 2010 and has been increasingly prevalent. Variant 2 possesses an A134T mutation and was present early in the pandemic, reaching a peak in 2010 and decreasing over time. Variant 3 is a double mutant (D222E and P297S) that appeared in late 2009, reaching its maximum in 2010 but declined afterward. These variants were interesting to study because the majority of the mutations are localized in or near the RBS. Variant 4, which has an E374K mutation, was present from the beginning of the pandemic and has dominated in the current influenza viruses; this mutation is localized near the fusion peptide in the stem of HA.

**Table 1:** Frequency of mutations in the amino acid sequence of Hemagglutinin (HA) from the influenza A(H1N1)pdm09 virus.

Collection date (year)^1^	Number of sequences	Variant 1 (%)		Variant 2 (%)	Variant 3 (%)		Variant 4 (%)
		S143G	S185T	A134T	D222E	P297S	E374K
**2009a**	1158	0.2	0.0	0.5	1.0	0.0	0.1
**2009b**	1607	0.1	0.0	0.4	**7.1** 2	3.1	19.8
**2010–2011**	200	9.5	11.5	**7.0**	6.5	**7.0**	50.5
**2011–2012**	530	**31.1**	**72.9**	1.9	0.2	0.0	**87.8**

1 2009a represents sequences from January-June 2009 period; 2009b represents sequences from the July-December 2009 period.

2 Highest frequencies are shown in bold.

Stability of the simulations was monitored through the RMSD, radius of gyration of protein backbone and root mean square fluctuation (RMSF) per amino acid. Stabilization of the systems started at approximately 800 picoseconds, and the simulation systems were fully equilibrated in the first nanosecond of the simulation (Figures S1 and S2). RMSF values (Figure S3) are similar for all the simulated systems, indicating that no major conformational changes occurred.

### Analysis of Molecular Dynamics Simulations

#### Variant 1 (S143G, S185T)

**Figure 1:**
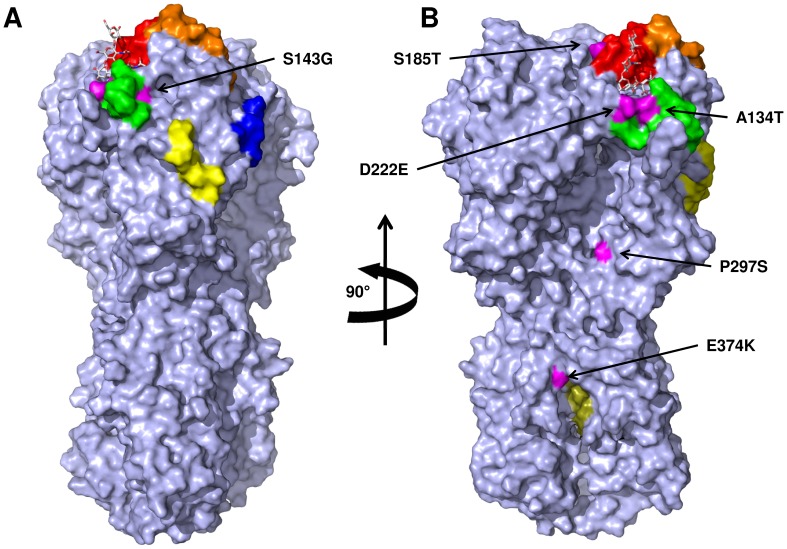
Surface representation of the Hemagglutinin (HA) form the influenza A(H1N1)pdm09 virus. The human receptor is shown as sticks. Mutations analyzed in this study are shown in magenta; antigenic sites are colored as follows: Ca1, blue; Ca2, green; Cb, yellow; Sa, orange, and Sb, red. The fusion peptide is colored olive green. Panel B represents the HA rotated 90° in the vertical axis.

Although mutation S143G has been reported since 2009, mutant S185T was not reported until 2010. Variant 1 rapidly became dominant, and in 2012, 29.4% of sequences corresponded to this variant. The mutations are localized in antigenic sites surrounding the RBS (Figure 1); S143G is localized at antigenic site Ca2, and S185T is within antigenic site Sb.

**Figure 2:**
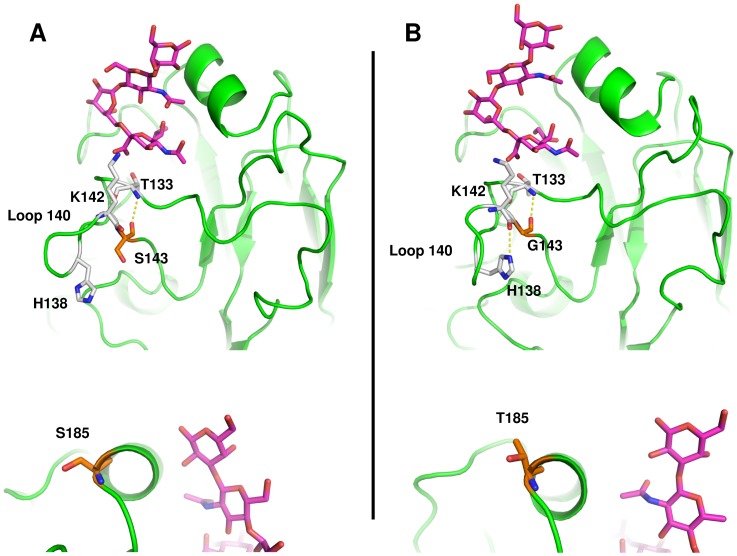
Conformational changes observed in variant 1. A, interactions of S143 in wild-type HA; B, interactions of S143G in variant 1; C, S185 in wild-type HA; D, S185T in variant 1. Amino acids surrounding position 138 are illustrated as sticks; the receptor is depicted as magenta sticks. Interactions with the receptor are essentially the same as the wild-type for variant 1. A displacement of loop 140 can be seen in variant 1, which allows H138 to interact with the main chain of K142 (B). Mutation S185T does not change interactions within the protein (D).

Amino acid 143 is localized in a loop protruding from the globular head of HA that involves residues 136–143 (loop 140). Analysis of molecular dynamics simulations showed that both the wild-type S143 and the mutant version G143 made stable hydrogen bonds through their main chain with T133 (Figure 2), which in turn was in contact with the sialic acid moiety of the receptor. Interestingly, removal of the bulky side chain in S143G rendered important rearrangements in the loop. Because of this movement we decided to extend the simulation of this variant as well as the wild-type HA to get a simulation of 10 nanoseconds. As the simulation progressed, a histidine residue (H138) moved into loop 140, establishing hydrogen bonds with the main chain of K142 (Figure 2A and S4). Analysis of solvent accessible surface area (SASA) showed that the histidine residue became less accessible to the solvent at approximately 5.5 nanoseconds, and continued in that state throughout the simulation (Figure S5). Thus mutation S143G changed the conformation of antigenic site Ca2 by hindering the histidine residue into loop 140. Along with the conformational change, this mutation caused a change in the local electrostatic potential (Figure S6). Regarding mutant S185T, the substitution did not create or eliminate interactions because in this position, the side chain is completely exposed with the solvent and rotates freely (Figure 2B).

This variant apparently did not alter the binding of the receptor because no evident conformational changes within the RBS were detected during simulations, and the binding free energy analysis (Table 2) showed no significant change in affinity for the receptor (–7.92 for the wild-type vs. –7.96 kcal/mol for Variant 1).

**Table 2:** Average binding free energies of protein-ligand complexes.

Variant	Binding free energy (kcal/mol)
**Wild-type**	–7.92
**S143G-S185T**	–7.96
**A134T**	–9.51
**D222E-P297S**	–8.56

#### Variant 2 (A134T)

**Figure 3:**
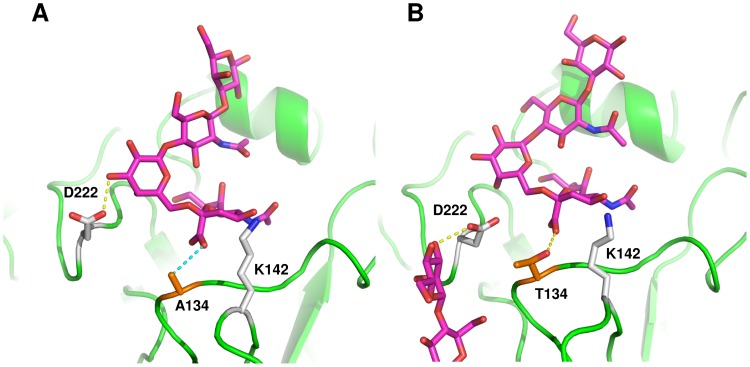
Effect of mutation S134T on receptor binding. A, wild-type HA; B, S134T. Protein is shown as green ribbons. Amino acids surrounding position 134 are illustrated as sticks; receptor and carbohydrate residues are depicted in magenta sticks. Mutation S134T modifies interactions of HA with the receptor by establishing hydrogen bonds between T134 and sialic acid, and by losing interactions between galactose and D222; instead, D222 in mutant S134T interacts with glycosylations originating at N87.

This mutant appeared to have a slight increase in frequency in 2010 but rapidly declined to levels below 1% by 2012. Residue 134 is located in the RBS near the antigenic site Ca2 (Figure 1); this residue interacted with the carboxyl group of sialic acid. In the wild-type HA, A134 made van der Waals contacts with K142 and was localized at 0.35 nm from the sialic acid carboxyl group. The A134T mutant established hydrogen bonds with sialic acid through the hydroxyl group of threonine (Figure 3A). At the beginning of the simulation, the threonine hydroxyl group was oriented away from the sialic acid carboxyl group (average distance, 0.48 nm), but as the simulation progressed, a rotation in the side chain placed the hydroxyl group near the sialic acid carboxyl group (avg. distance 0.29 nm) (Figure 3B; Figure S7). Rotation of T134 pushed the sialic acid receptor deeper into the RBS, thus losing polar interactions between D222 and the galactose residue (Figure S8). The methyl group of T134 made van der Waals contacts with the β carbon of D222 and sugar residues from nearby glycosylations originating at N87. These new contacts favored receptor binding by –1.59 kcal/mol compared with the wild-type HA (Table 2).

#### Variant 3 (D222E, P297S)

**Figure 4:**
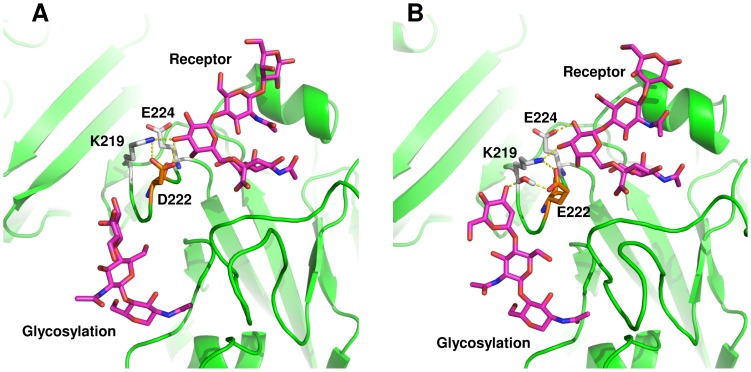
Effect of D222E in receptor binding. A, wild-type HA; B, D222E mutant. Protein is shown as green ribbons. Amino acids surrounding position 222 are illustrated as sticks; receptor and carbohydrate residues are depicted as magenta sticks. In the wild-type HA, D222 forms a triad with K219 and E224 that interact with galactose from the human receptor. In D222E, only K219 and E224 interact with galactose, while E222 interacts through water-mediated hydrogen bonds with nearby glycosylations originating at N87.

In this variant, mutation D222E is located in the RBS within antigenic site Ca2, whereas P297S is localized in the upper stem portion of HA (Figure 1). Analysis of the molecular dynamics simulations showed that in wild-type HA, D222 established stable polar contacts with galactose from the receptor and with K219 (Figure 4A). In D222E, the extra carbon in the side chain of E222 caused the glutamate’s carboxyl group to move away from the RBS, rendering water-mediated polar contacts with a terminal mannose from glycosylations originating from N87 and van der Waals contacts with galactose from the receptor (Figure 4B). Rearrangement of the receptor within the RBS made E224 sufficiently close to contact galactose at O2 (Figure 4B; Figure S9). These interactions favored binding free energy by –0.64 kcal/mol in comparison with wild-type HA (Table 2).

Regarding mutation P297S, we could not deduce a change in interactions during the molecular dynamics simulations. The mutation is localized in the upper side of the HA stem portion in a solvent-accessible portion of HA; both the wild-type and the mutant maintained their interactions during molecular dynamics simulations.

#### Variant 4 (E374K)

**Figure 5:**
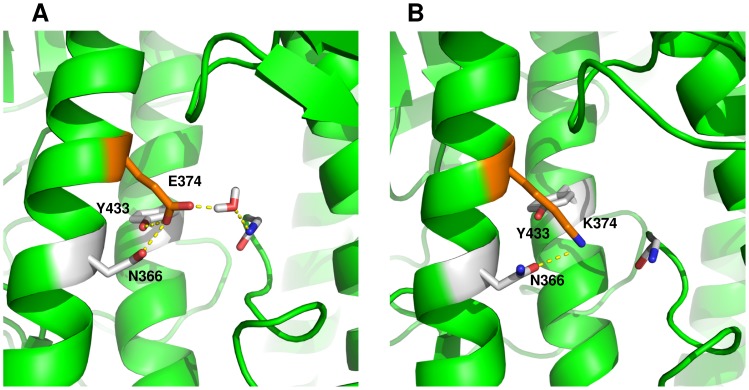
Destabilizing effect of mutation E374K. A, wild-type HA; B, E374K mutant. Protein is shown as green ribbons. Amino acids surrounding position 374 are illustrated as sticks. In the wild-type HA, E374 interacts with Y433 and N366 and with the fusion peptide through water-mediated hydrogen bonds. In E374K, the lysine residue interacts only with the oxygen from the amide group of N366.

This variant bears a mutation in a pocket formed in the subunit interface that harbors the fusion peptide (Figure 1). Stability analysis performed with I-Mutant and CUPSAT suggested that the E374K mutation in HA would have a destabilizing structural effect (I-Mutant ΔΔG = −0.65 kcal/mol; CUPSAT ΔΔG = −1.58 kcal/mol). This variant did not reach stability within the first 5 nanoseconds of simulation (Figure S1), so we decided to extend the simulation of this variant to 10 nanoseconds. Molecular dynamics simulations of wild-type HA showed that E374 made intra-chain contacts with N366 (Figure S10) and Y433 (Figure S11) and water-mediated hydrogen bonds with the N-terminal portion of the fusion peptide. Interactions with N366 were established mainly through nitrogen from the amide group, thus exposing the amide’s oxygen to the solvent. In E374K, the longer hydrocarbon side chain of lysine eliminated interactions with Y433 (Figure S11), while polar contacts with N366 shifted to the amide’s oxygen (Figure S10), exposing the amino group to the solvent (Figures 5A and 5B).

**Figure 6:**
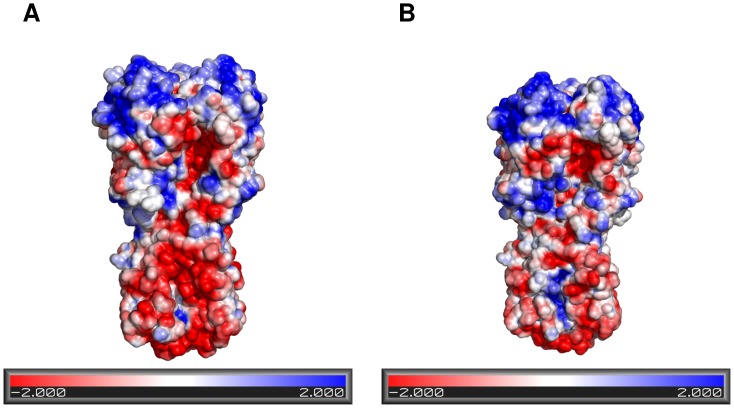
Surface electrostatic potential in wild-type and E374K HA. A, wild-type HA; B, E374K mutant. Electrostatic potential was colored into the surface structure. Red-Blue gradient was set from –2 to +2 kT/e. The E374K mutant exhibits a decrement in negative charge distribution in the stem portion of HA because the carboxyl group from glutamic acid is replaced by an amino group from lysine and interactions with N366 changed, thus exposing the amino group of N366 to the solvent.

The positive charge of K391 and the exposure of the amide’s amino group from N366 drastically changed the charge distribution in HA. As can be observed in Figures 6A and 6B, wild-type HA has a marked charge distribution, with the lower part of HA predominantly negative and the top, positive; however, in E374K, the protein loses negative electrostatic potential and gains positive potential inside the fusion peptide pocket.

## Discussion

Variation in HA is expected as a consequence of two main factors: Selective pressure associated with immune response, mainly when the vaccine was available worldwide, and adaptation to the human host, in which mutations that increased viral transmission were positively selected. We studied four variants of the HA of influenza virus that emerged at the beginning of the 2009 influenza pandemic, but had different fates as the pandemic evolved. Three of the variants contain mutations located in or near the RBS, while one had a mutation in the fusion peptide pocket in the HA stem portion.

Variant 1 has become dominant in current influenza viruses. This variant has been reported previously [31], and was associated with a severe form of influenza in infected patients. Our data showed that mutations in variant 1 could confer an evolutionary advantage to influenza viruses by evading the immune response. Despite being localized near the RBS, these mutations did not modify the shape of the RBS nor did they disrupt interactions between HA and the receptor. Mutation S143G created a rearrangement in loop 130–140, altering the conformation and charge distribution of antigenic site Ca2 by hindering a histidine residue located at the tip of the loop. On the other hand, residue position 185 is localized in an α-helix that delimitates the RBS. Contrary to what happened with other mutations, S185T caused no change in interactions with nearby amino acids. Thus, we assumed that a likely cause that would positively select mutation S185T was immune recognition avoidance. It has been demonstrated previously that point mutations within antigenic sites may arise as a consequence of immune pressure, and do not necessarily alter protein function. An example can be seen in O’Donnell *et al.* [32] 2012 (doi:10.1128/mBio.00120-12) in which the escape mutant K157N had characteristics similar to the wild type HA, although it was no longer recognized by antibodies.

Mutations in variants 2 and 3 created slight changes in the RBS that increased the number of interactions with the receptor, lowering, in this case, the binding free energy of the receptor-HA complex. In the case of variant 3, polymorphism at position 222 has been extensively studied due mainly to mutation D222G, which has been associated with severe influenza as a consequence of change in the preference of sialic acid receptors [33]–[37]. Consistent with our results, mutation D222G possesses increased affinity for α2,6 receptors compared with D222 [33].

Contrary to what was expected, mutations that increased receptor binding affinity were at selective disadvantage, because these variants arose and perished shortly after reaching their maximum prevalence in 2010. We think that these mutations, despite being advantageous by increasing infectivity, do not change the antigenic mosaic of HA sufficiently to avoid recognition by the immune system; thus, these were easily displaced as the human population mounted an immune response against the virus. Another possibility to explain the demise of variants 2 and 3 is that HA and NA activity must be balanced for the influenza virus to infect cells properly and to disseminate to other hosts [38], [39]. It has been found that the influenza A(H1N1)pdm2009 virus has low-affinity HA and weakly active NA [39]; therefore, a mutation that increases the binding of HA to human receptors would hamper virus transmissibility.

Variant 4 is another example of a mutation that has become predominant in current influenza viruses. Mutation E374K is located in a pocket harboring the fusion peptide. Our results showed that the mutation causes a decrease in intramolecular interactions that, in conjunction with a shift in the electrostatic potential in the stem portion of HA to a more positive value, could have a destabilizing effect on HA. This effect was also confirmed with structural stability analysis performed with I-Mutant and CUPSAT. The stabilization or destabilization of HA and its relationship with HA activity as a consequence of mutations has been explored in H3 [40], in which stabilizing mutations led to a decrease in the pH required for activation of HA. In the case of the H5 subtype, changes that destabilize HA increase the pH of activation, hence virulence in mallards [41]. Thus, we propose that the E374K mutation may yield a more unstable HA, with an increased pH of activation, and hence an increased capability to infect cells. This could increase the virulence or transmissibility of the virus, explaining the growing prevalence of this mutation throughout the 2009 pandemic.

### Conclusions

We studied mutants that arose from the beginning of the pandemic, including some that predominate in current circulating viruses. We found that mutants that alter the balance between HA avidity and NA activity are detrimental for the virus. On the other hand, the variants that prevailed as the pandemic evolved were mainly escape mutants that should be studied more and taken into account for vaccine development. One mutant is expected to increase virus fitness by destabilizing the HA, thus allowing for more extensive distribution in human population. These results point to interesting hypotheses of how these mutations affect the structure and function of the hemagglutinin and these hypotheses can now be tested further with biochemical analysis.

## Supporting Information

Figure S1 **Root-mean-square deviation of the protein backbone.** The root-mean-square deviation (RMSD) reflected the stability of the protein as the simulation progressed. Stabilization of the proteins starts within the first nanosecond of simulation. WT, variant 1 and variant 4 were simulated for additional 5 nanoseconds to allow for protein stabilization. Color code: WT, blue; variant 1, red; variant 2, green; variant 3, yellow; variant 4, orange.
(PDF)

Figure S2 **Radius of gyration of the protein backbone.** In addition to root-mean-square deviation, equilibration of the simulation system was monitored through the radius of gyration (Rg) of the protein backbone, which is a measure of the compactness of the protein. Stabilization can be observed at approximately 1000 picoseconds. WT, variant 1 and variant 4 were simulated for additional 5 nanoseconds to allow for protein stabilization. Color code: WT, blue; variant 1, red; variant 2, green; variant 3, yellow; variant 4, orange.
(PDF)

Figure S3 **Root-mean-square fluctuation per amino acid.** The root-mean-square fluctuation (RMSF) shows flexible and rigid portions of the protein. Values are the average RMSF for each amino acid in the three monomers of HA. Flexible and rigid regions are the same for the wild type and all HA variants included in this study. Color code: WT, blue; variant 1, red; variant 2, green; variant 3, yellow; variant 4, orange.
(PDF)

Figure S4 **The S143G mutation facilitates interaction between H138 and K142 through hydrogen bonds.** Mutation S143G creates a void within loop 140 that is occupied by H138, causing a conformational change that draws together the side chain of H138 and the main chain of K142. The conformational change starts at approximately 5500 picoseconds. Hydrogen bonds were calculated with g_hbond, with distance an angle cut offs of 0.35 and 35, respectively. Color code: Gray, detection of hydrogen bonds in variant 1 (no hydrogen bonds were detected in the wild type hemagglutinin); distances between the side chain of H138 and the main chain of K142 are shown in yellow for the wild type and red for the variant 1 hemagglutinins.
(PDF)

Figure S5 **Solvent accesible surface area of the hydrophilic portion of H138.** The conformational change caused by mutation S134G in variant 1 causes partial hindering of H138 within loop 140, starting at approximately 5500 picoseconds. Color code: WT, blue; S143G, red.
(PDF)

Figure S6 **Displacement of H134 changed the shape and local charge distribution in variant 1.** Electrostatic potential was calculated for the initial conformation of variant 1 (left) and for the conformation at the eight nanosecond of simulation (right). The histidine residue moved into loop 140, changing the shape of loop 140 and also the local charge distribution. Solvent-accessible surface is shown at 40% transparency to improve visualization of H138, shown as sticks. Protein backbone is shown as cartoon. Pymol (www.pymol.org) and APBS [26] were used to generate this figure.
(PDF)

Figure S7 **Distance between the side chain of amino acid 134 and the carboxyl group of sialic acid.** During the simulation, A134 was at an average distance of 0.35 nm from the carboxyl group of sialic acid. In A134T, treoninés side chain rotates, placing its hydroxyl group at an average distance of 0.29 nm from the carboxyl group of sialic acid. Color code: WT, blue; A134T, red.
(PDF)

Figure S8 **Distance between D222 and galactosés O3 in the wild-type and variant 2 HAs.** During the simulation, D222 in the wild-type HA was at an average distance of 0.31 nm from galactosés O3, whereas for variant 2, the average distance was of 0.5 nm. Color code: WT, blue; variant 2, red.
(PDF)

Figure S9 **D224-Gal. Distance between D224 and galactosés O2 in the wild-type and variant 3 HAs.** During the simulation, D224 in the wild-type HA was at an average distance of 0.71 nm from galactosés O3, whereas for variant 3, the average distance was of 0.41 nm in the last two nanoseconds of simulation. Color code: WT, blue; variant 3, red.
(PDF)

Figure S10 **Distance between the side chain of amino acid 374 and N366.** During the simulation, E374 (wild-type HA) was at an average distance of 0.38 nm from the nitrogen of the amide group of N366. In E374K, the amino group of K374 remained at an average distance of 0.33 nm from the oxygen of the amide group of N366. Color code: WT, blue; A134T, red.
(PDF)

Figure S11 **Distance between amino acid 374 and Y433.** During the simulation, E374 (wild-type HA) was at an average distance of 0.31 nm from the hydroxyl group of Y433. In E374K, the amino group of K374 remained at an average distance of 0.50 nm from the hydroxyl group of Y433. Color code: WT, blue; A134T, red.
(PDF)

Table S1 **Amino acid substitution frequency for influenza A(H1N1)pdm09 hemagglutinin.** Substitution frequencies were grouped by year of isolation. Year 2009 was divided by semester (January-June had 1158 sequences, whereas July-December had 1607); sequences from viruses isolated in 2010 we grouped together (200 sequences), and the last group contained sequences from 2011 until November 2012 (530 sequences).
(XLSX)
