# Supplementary figures and images for: Analysis of Adaptation Mutants in the Hemagglutinin of the Influenza A(H1N1)pdm09 Virus
Source: PLoS One. 2013 Jul 23;8(7):e70005. doi: 10.1371/journal.pone.0070005 (PMC3720954; doi:10.1371/journal.pone.0070005)

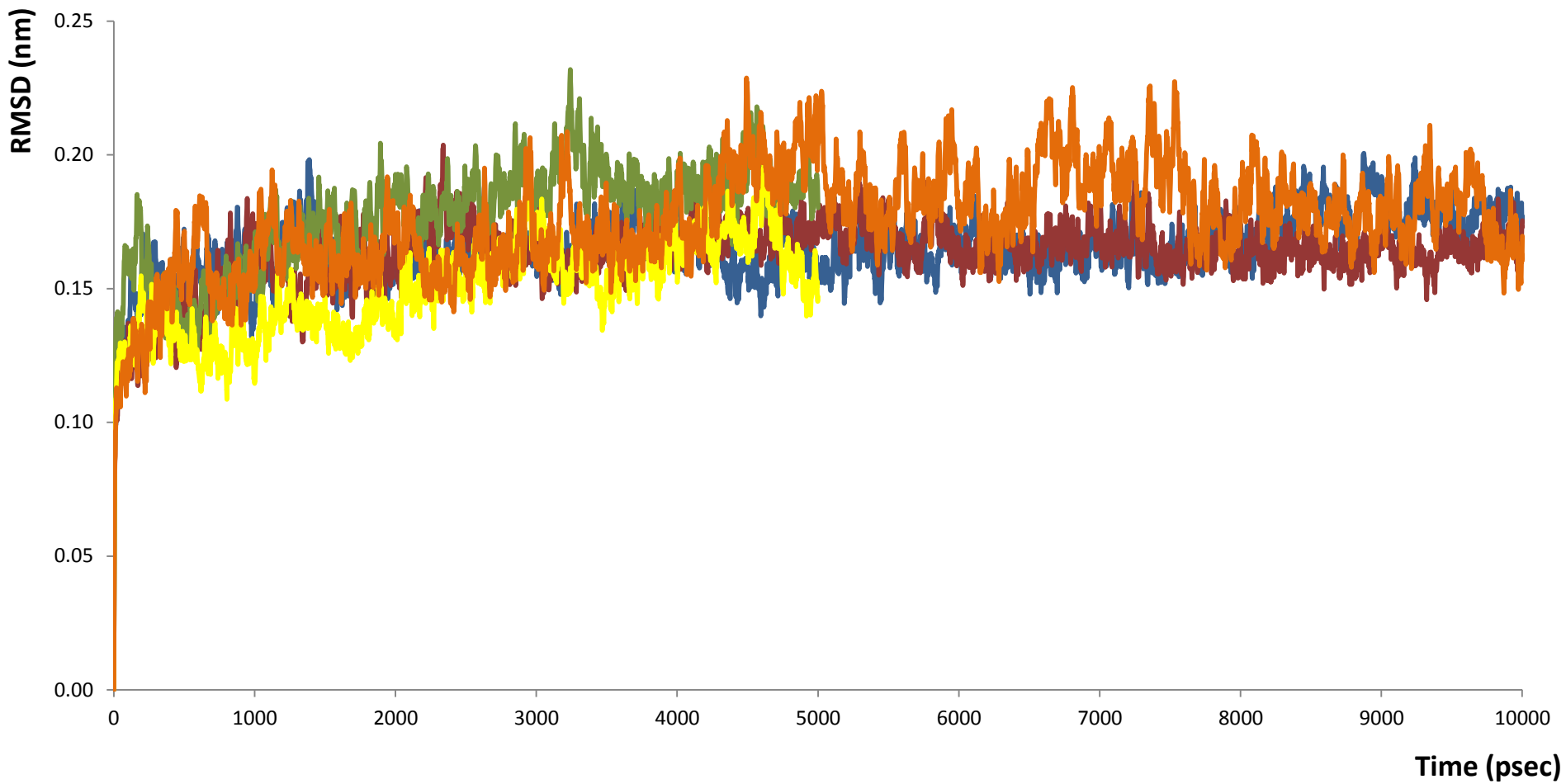

Supplement: Figure S1 — Root-mean-square deviation of the protein backbone. The root-mean-square deviation (RMSD) reflected the stability of the protein as the simulation progressed. Stabilization of the proteins starts within the first nanosecond of simulation. WT, variant 1 and variant 4 were simulated for additional 5 nanoseconds to allow for protein stabilization. Color code: WT, blue; variant 1, red; variant 2, green; variant 3, yellow; variant 4, orange. (PDF) [file pone.0070005.s001.pdf]

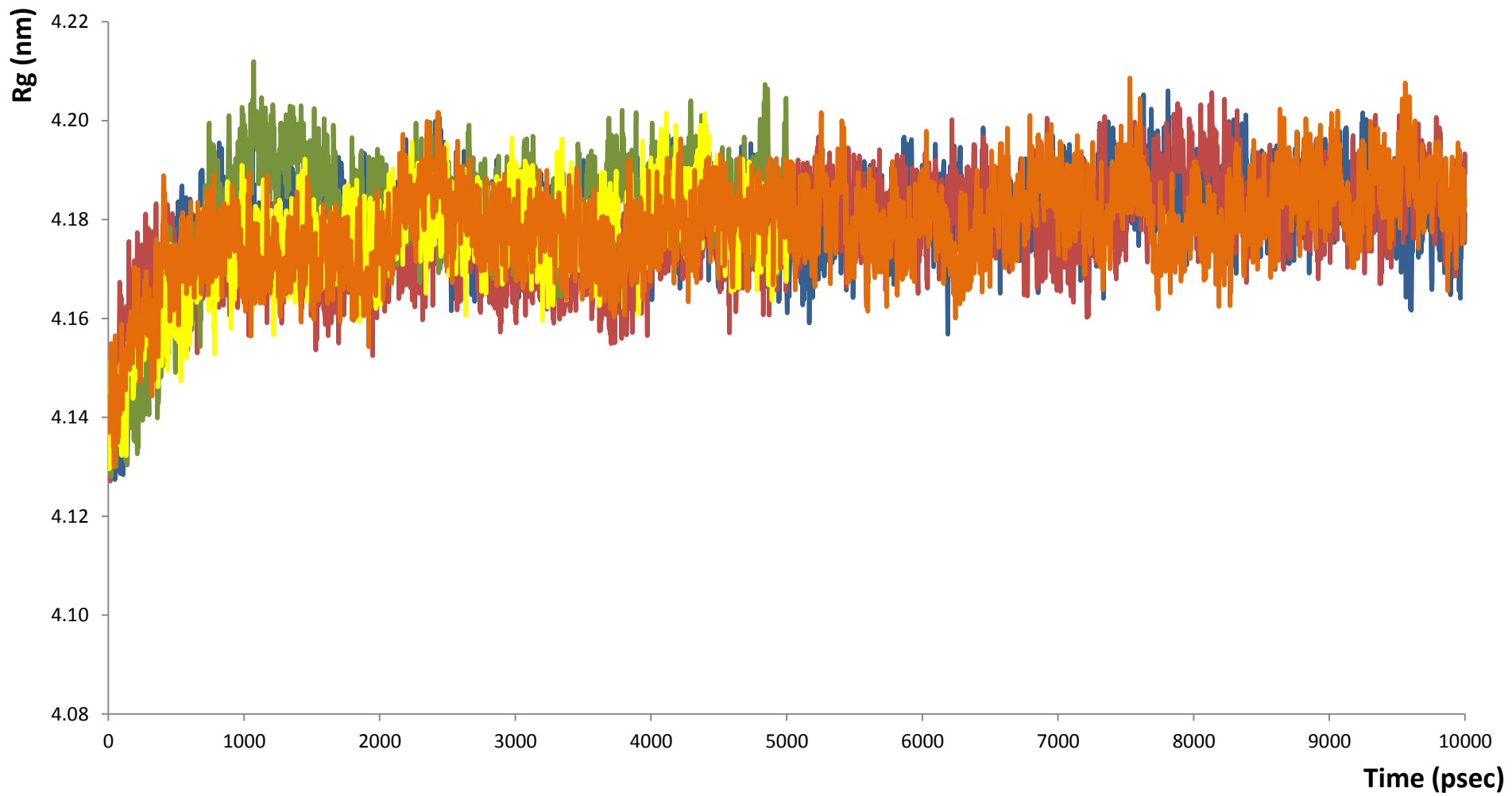

Supplement: Figure S2 — Radius of gyration of the protein backbone. In addition to root-mean-square deviation, equilibration of the simulation system was monitored through the radius of gyration (Rg) of the protein backbone, which is a measure of the compactness of the protein. Stabilization can be observed at approximately 1000 picoseconds. WT, variant 1 and variant 4 were simulated for additional 5 nanoseconds to allow for protein stabilization. Color code: WT, blue; variant 1, red; variant 2, green; variant 3, yellow; variant 4, orange. (PDF) [file pone.0070005.s002.pdf]

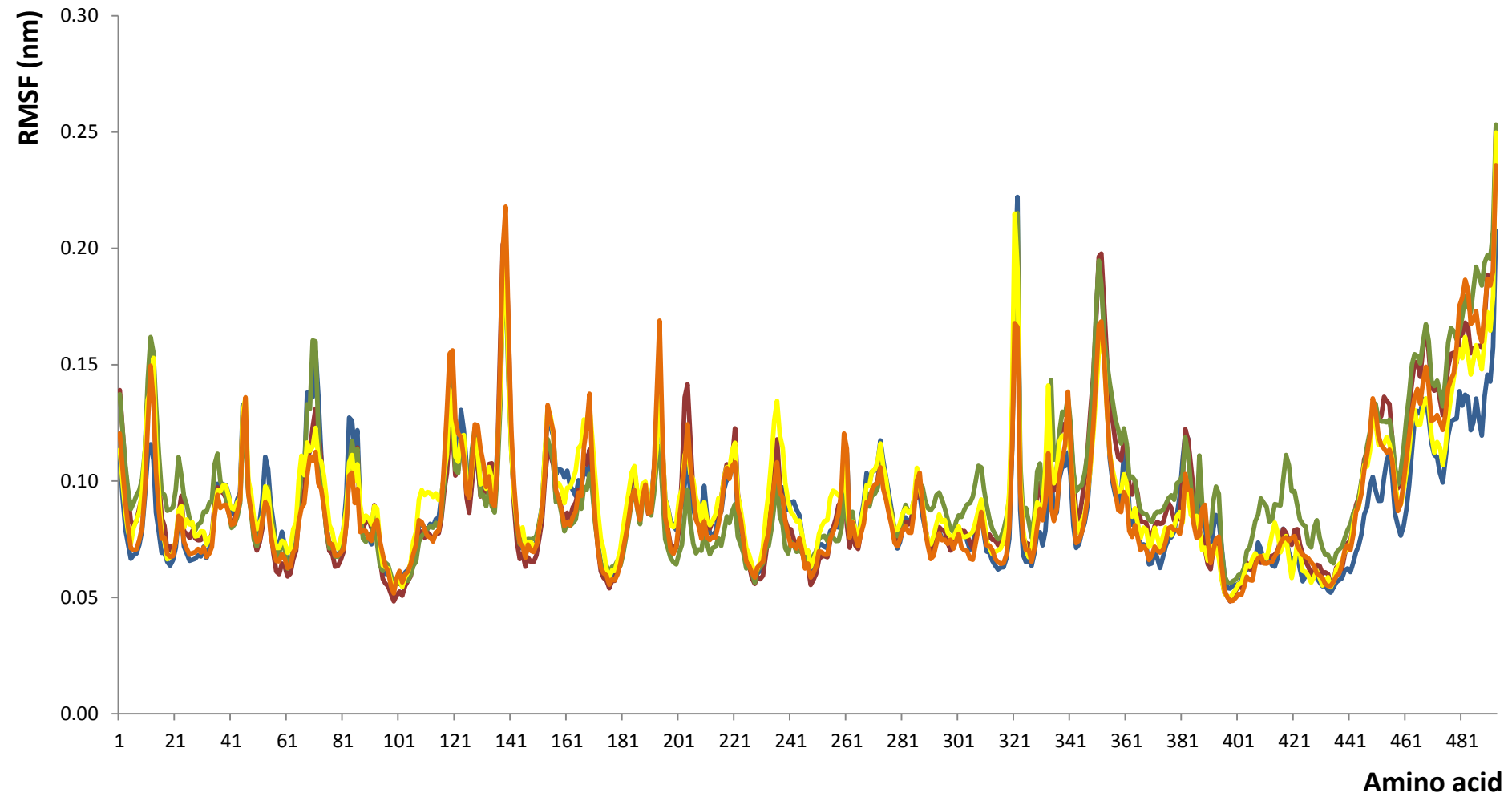

Supplement: Figure S3 — Root-mean-square fluctuation per amino acid. The root-mean-square fluctuation (RMSF) shows flexible and rigid portions of the protein. Values are the average RMSF for each amino acid in the three monomers of HA. Flexible and rigid regions are the same for the wild type and all HA variants included in this study. Color code: WT, blue; variant 1, red; variant 2, green; variant 3, yellow; variant 4, orange. (PDF) [file pone.0070005.s003.pdf]

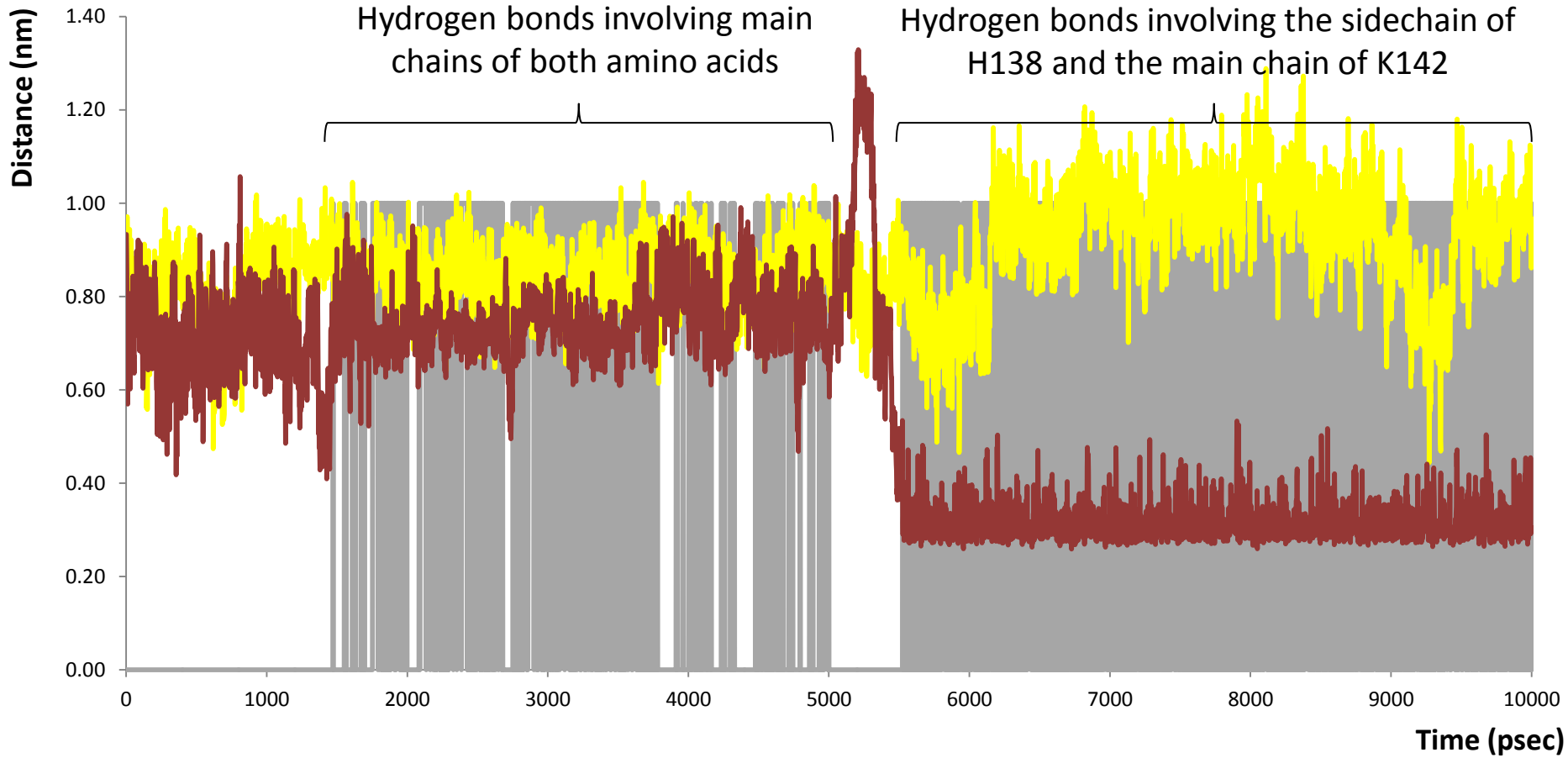

Supplement: Figure S4 — The S143G mutation facilitates interaction between H138 and K142 through hydrogen bonds. Mutation S143G creates a void within loop 140 that is occupied by H138, causing a conformational change that draws together the side chain of H138 and the main chain of K142. The conformational change starts at approximately 5500 picoseconds. Hydrogen bonds were calculated with g_hbond, with distance an angle cut offs of 0.35 and 35, respectively. Color code: Gray, detection of hydrogen bonds in variant 1 (no hydrogen bonds were detected in the wild type hemagglutinin); distances between the side chain of H138 and the main chain of K142 are shown in yellow for the wild type and red for the variant 1 hemagglutinins. (PDF) [file pone.0070005.s004.pdf]

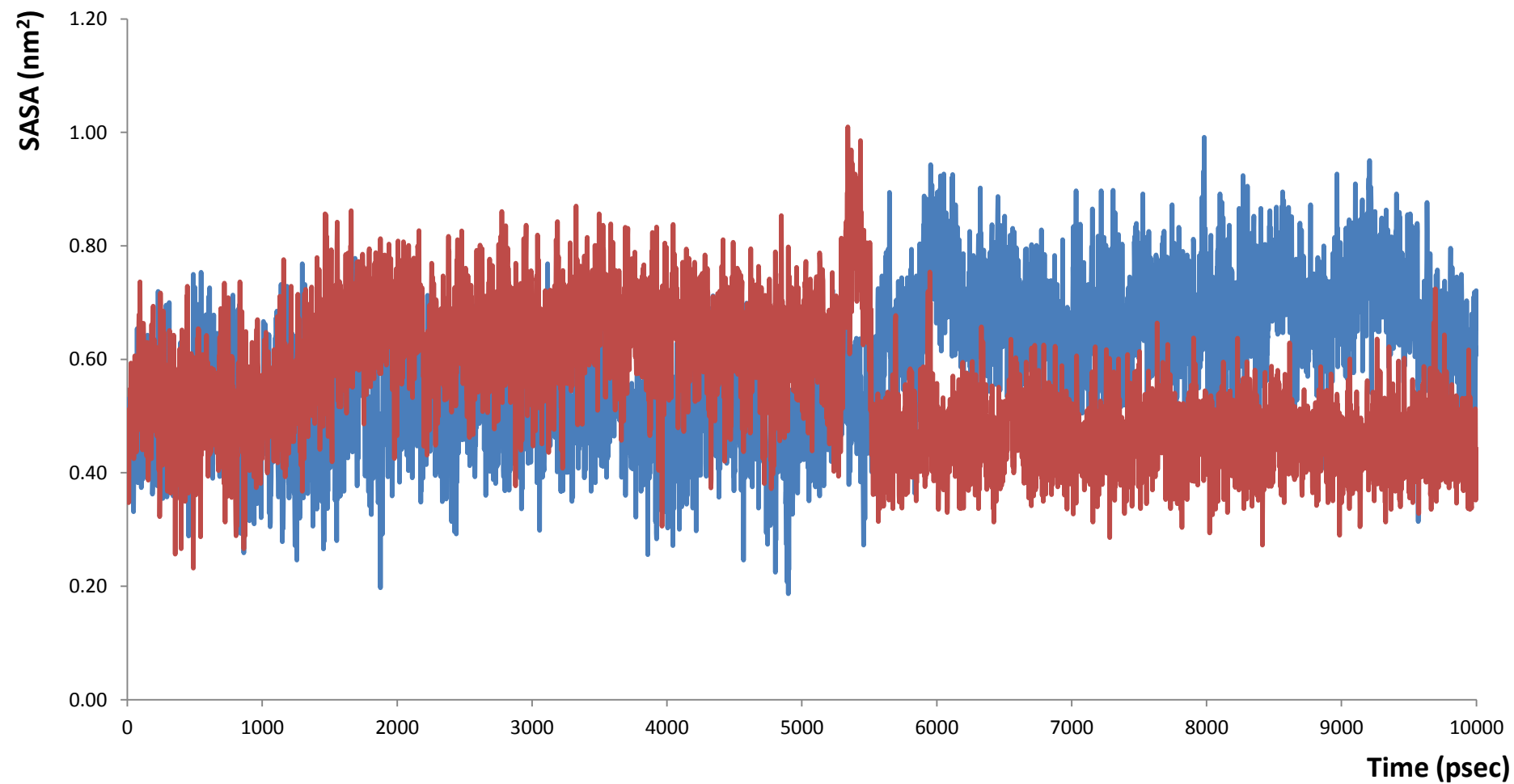

Supplement: Figure S5 — Solvent accesible surface area of the hydrophilic portion of H138. The conformational change caused by mutation S134G in variant 1 causes partial hindering of H138 within loop 140, starting at approximately 5500 picoseconds. Color code: WT, blue; S143G, red. (PDF) [file pone.0070005.s005.pdf]

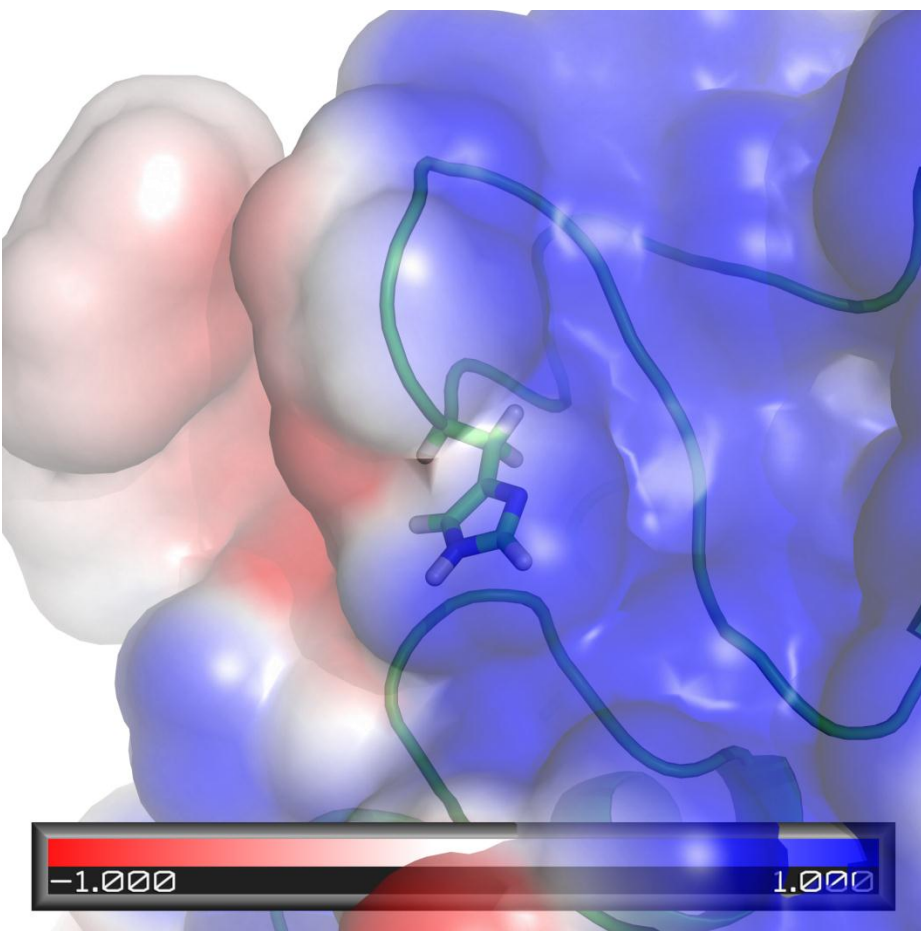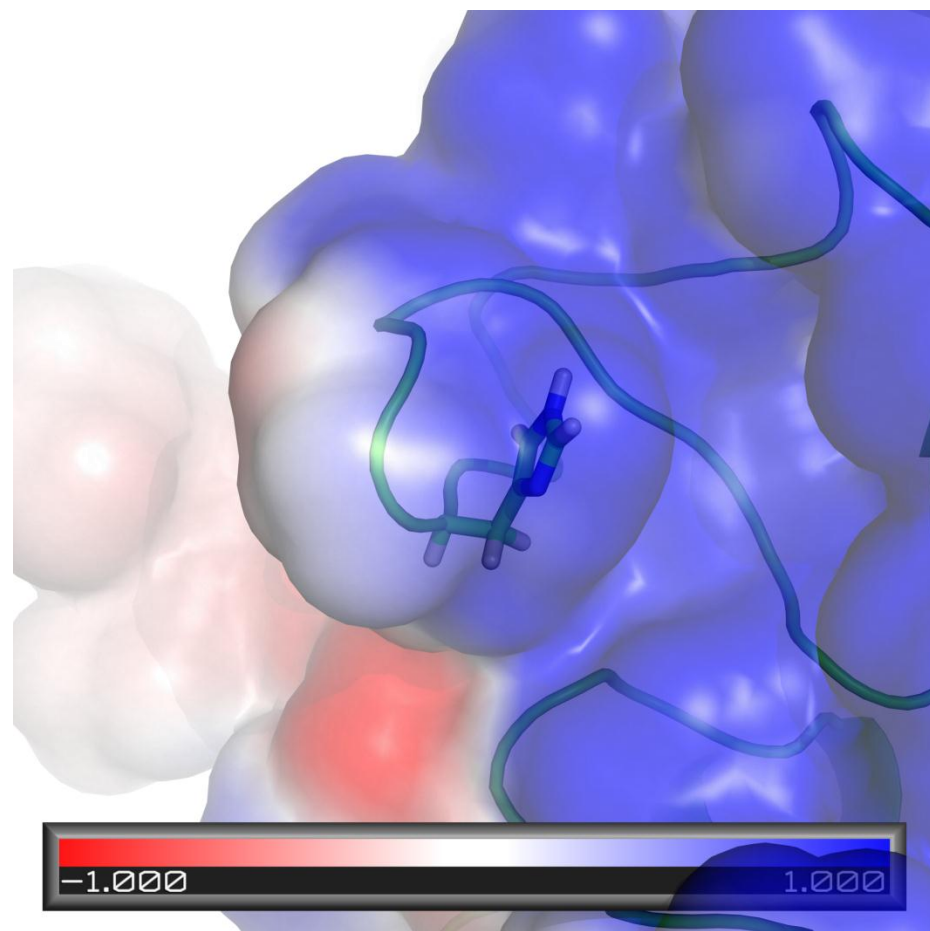

Supplement: Figure S6 — Displacement of H134 changed the shape and local charge distribution in variant 1. Electrostatic potential was calculated for the initial conformation of variant 1 (left) and for the conformation at the eight nanosecond of simulation (right). The histidine residue moved into loop 140, changing the shape of loop 140 and also the local charge distribution. Solvent-accessible surface is shown at 40% transparency to improve visualization of H138, shown as sticks. Protein backbone is shown as cartoon. Pymol (www.pymol.org) and APBS [26] were used to generate this figure. (PDF) [file pone.0070005.s006.pdf]

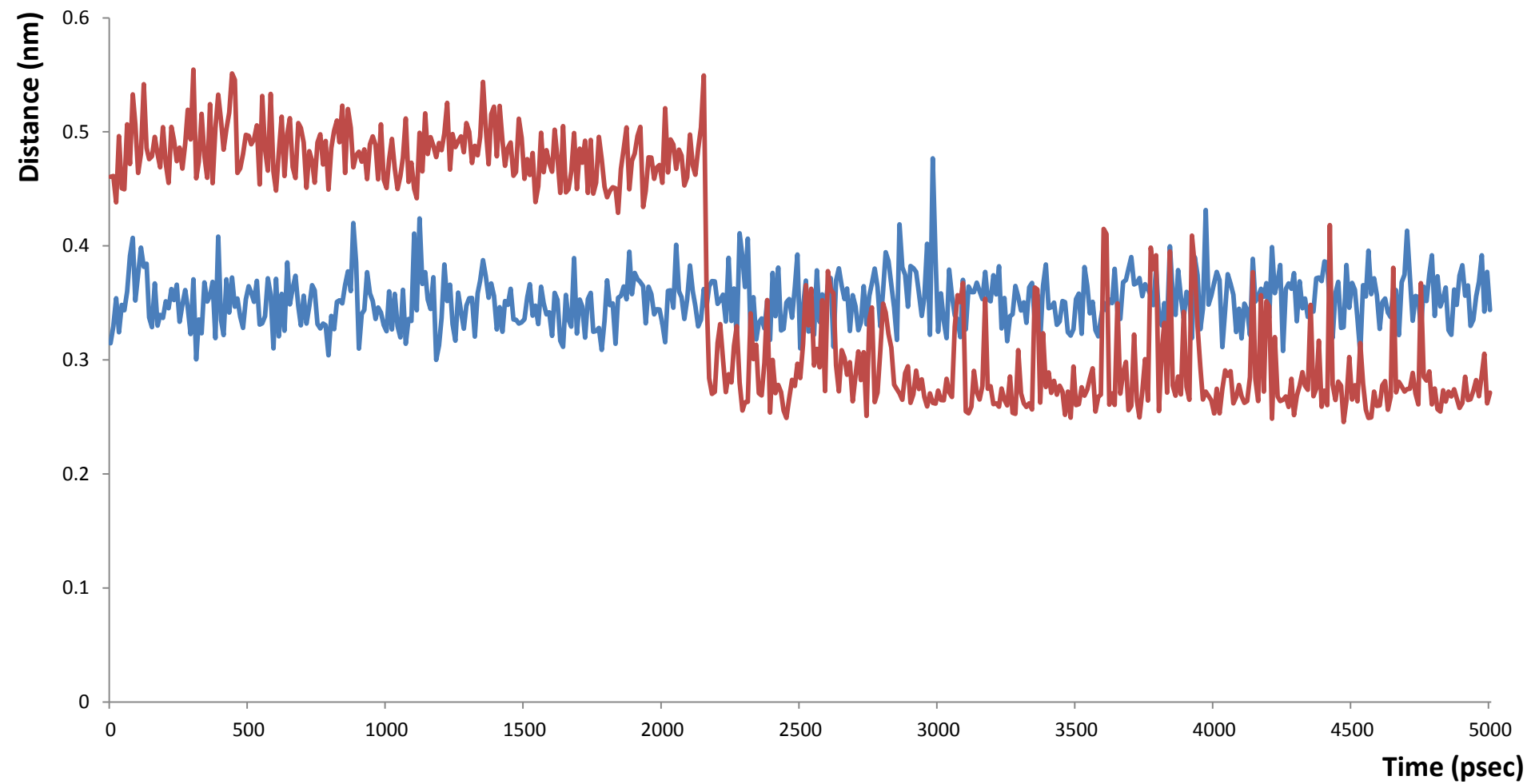

Supplement: Figure S7 — Distance between the side chain of amino acid 134 and the carboxyl group of sialic acid. During the simulation, A134 was at an average distance of 0.35 nm from the carboxyl group of sialic acid. In A134T, treoninés side chain rotates, placing its hydroxyl group at an average distance of 0.29 nm from the carboxyl group of sialic acid. Color code: WT, blue; A134T, red. (PDF) [file pone.0070005.s007.pdf]

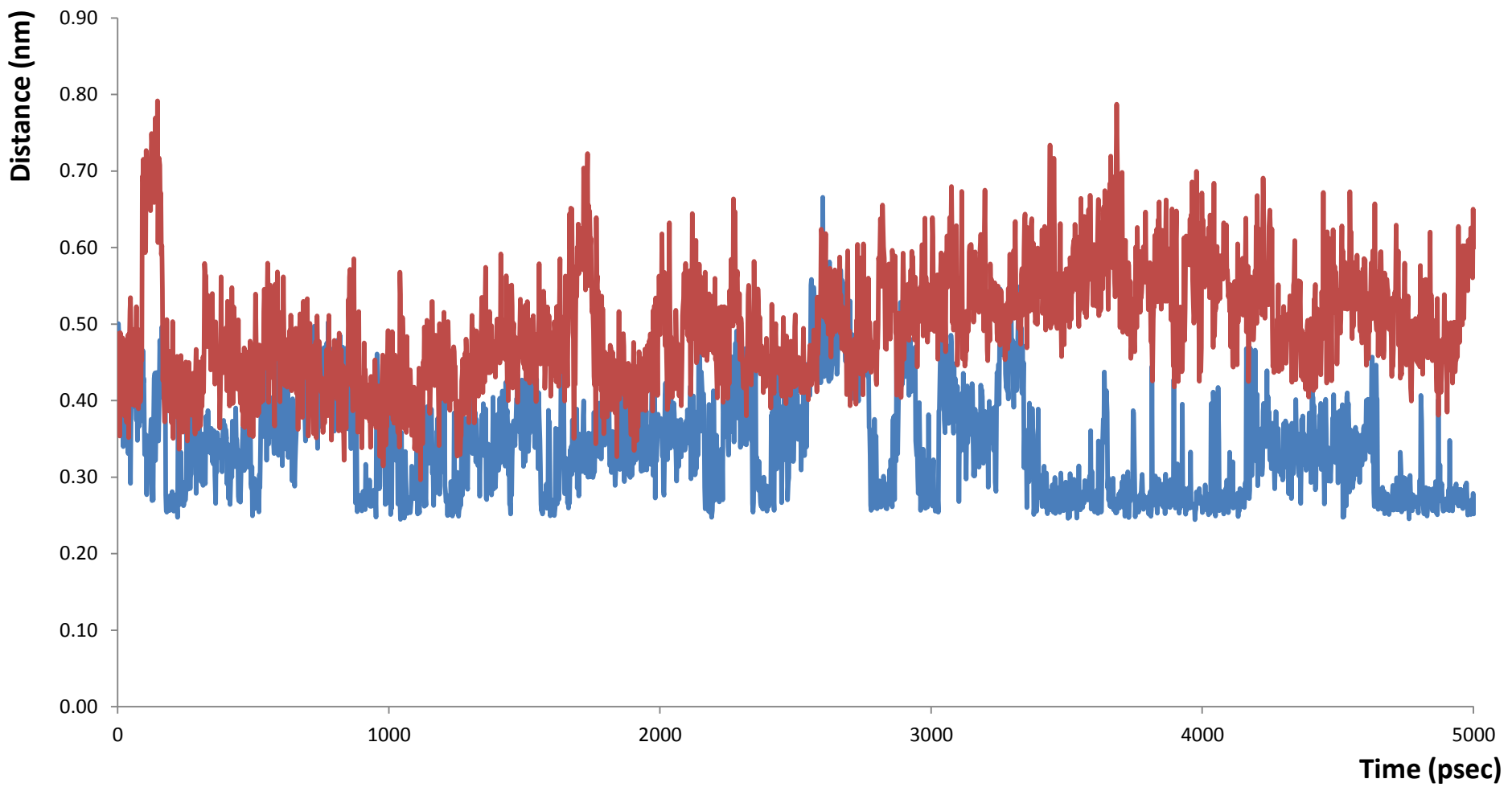

Supplement: Figure S8 — Distance between D222 and galactosés O3 in the wild-type and variant 2 HAs. During the simulation, D222 in the wild-type HA was at an average distance of 0.31 nm from galactosés O3, whereas for variant 2, the average distance was of 0.5 nm. Color code: WT, blue; variant 2, red. (PDF) [file pone.0070005.s008.pdf]

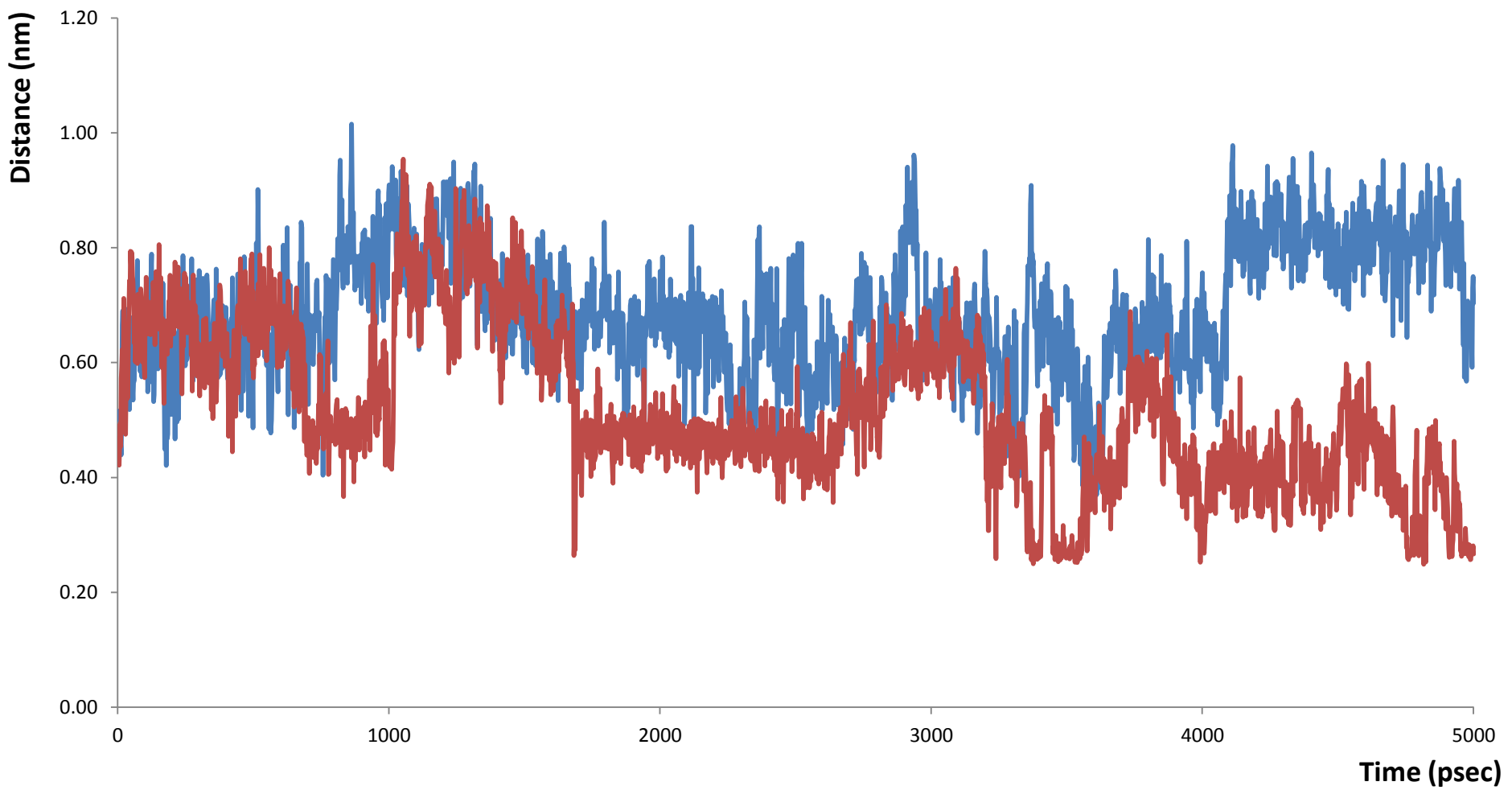

Supplement: Figure S9 — D224-Gal. Distance between D224 and galactosés O2 in the wild-type and variant 3 HAs. During the simulation, D224 in the wild-type HA was at an average distance of 0.71 nm from galactosés O3, whereas for variant 3, the average distance was of 0.41 nm in the last two nanoseconds of simulation. Color code: WT, blue; variant 3, red. (PDF) [file pone.0070005.s009.pdf]

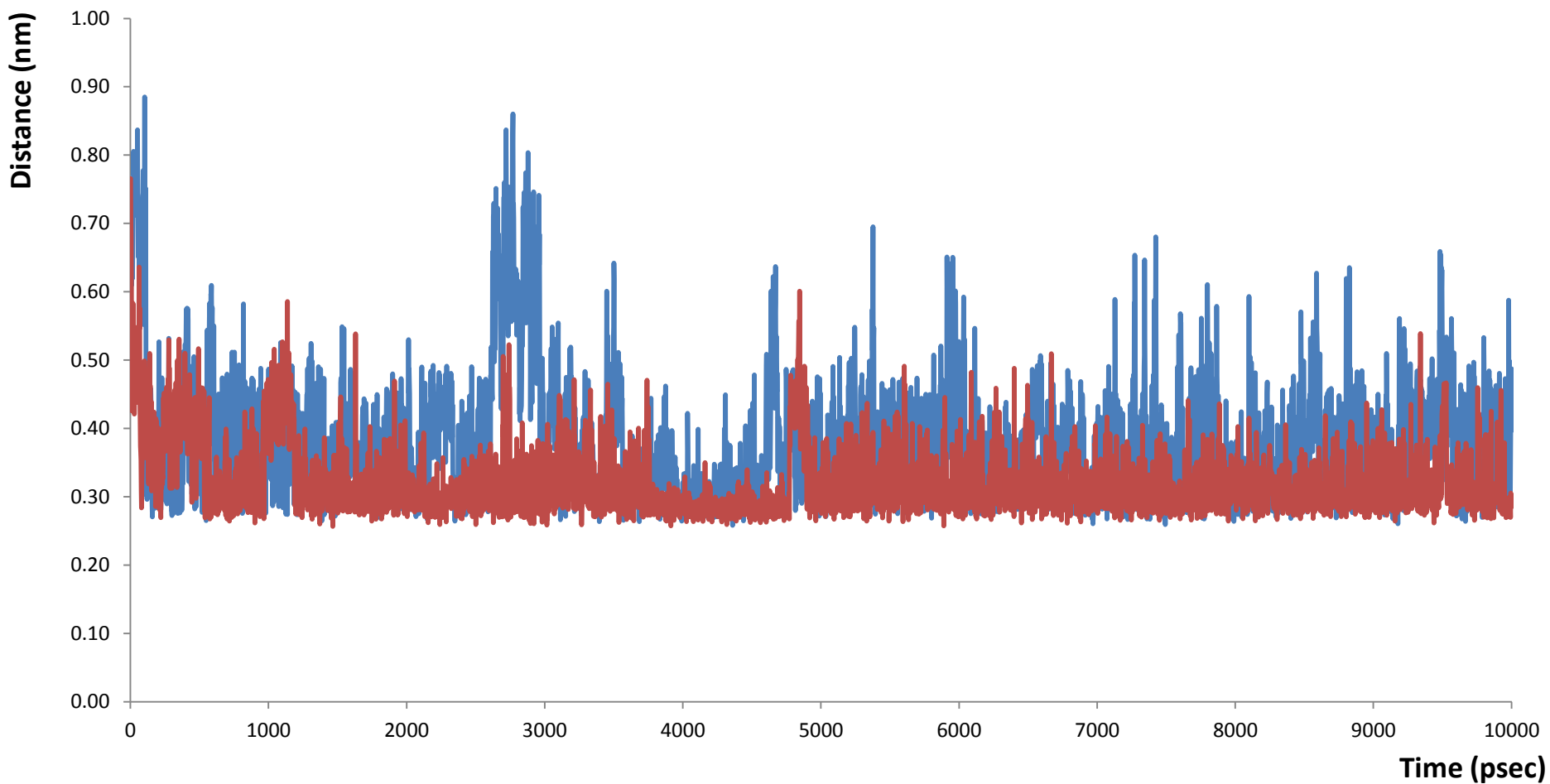

Supplement: Figure S10 — Distance between the side chain of amino acid 374 and N366. During the simulation, E374 (wild-type HA) was at an average distance of 0.38 nm from the nitrogen of the amide group of N366. In E374K, the amino group of K374 remained at an average distance of 0.33 nm from the oxygen of the amide group of N366. Color code: WT, blue; A134T, red. (PDF) [file pone.0070005.s010.pdf]

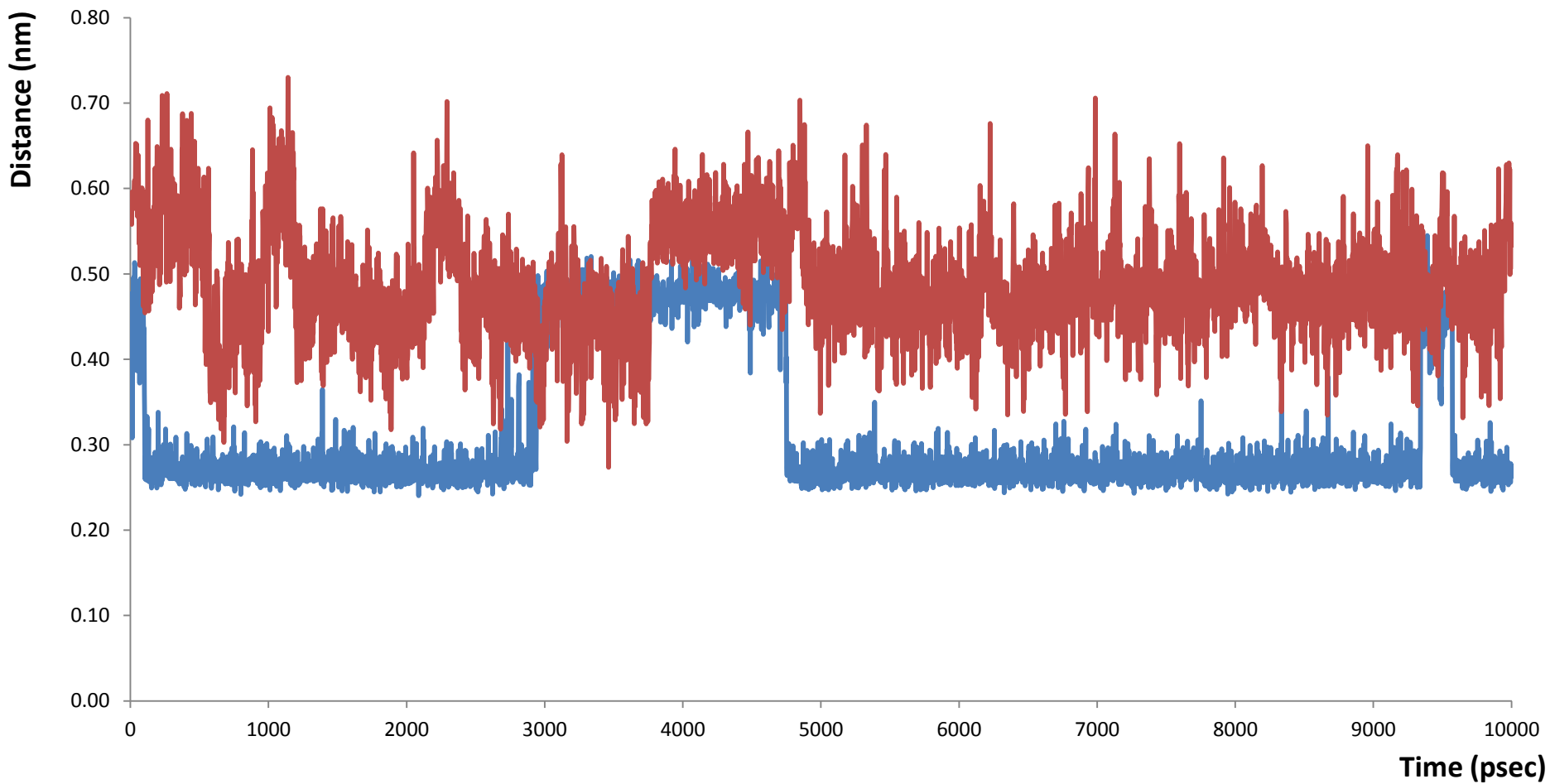

Supplement: Figure S11 — Distance between amino acid 374 and Y433. During the simulation, E374 (wild-type HA) was at an average distance of 0.31 nm from the hydroxyl group of Y433. In E374K, the amino group of K374 remained at an average distance of 0.50 nm from the hydroxyl group of Y433. Color code: WT, blue; A134T, red. (PDF) [file pone.0070005.s011.pdf]
